# The funding landscape for HIV in Asia and the Pacific

**DOI:** 10.7448/IAS.18.1.20004

**Published:** 2015-11-16

**Authors:** Robyn M Stuart, Eric Lief, Braedon Donald, David Wilson, David P Wilson

**Affiliations:** 1 The Kirby Institute, University of New South Wales, Sydney, Australia; 2 Department of Mathematical Sciences, University of Copenhagen, Copenhagen, Denmark; 3 The Burnet Institute, Melbourne, Australia; 4 Global HIV/AIDS Program, The World Bank, Washington, DC, USA

**Keywords:** HIV, bilateral, multilateral, funding, Asia, Pacific

## Abstract

**Introduction:**

Despite recent and robust economic growth across the Asia-Pacific region, the majority of low- and middle-income countries in the region remain dependent on some donor support for HIV programmes. We describe the availability of bilateral and multilateral official development assistance (ODA) for HIV programmes in the region.

**Methods:**

The donor countries considered in this analysis are Australia, Canada, Denmark, France, Germany, Netherlands, Norway, Sweden, the United Kingdom and the United States. To estimate bilateral and multilateral ODA financing for HIV programmes in the Asia-Pacific region between 2004 and 2013, we obtained funding data from the Organisation for Economic Co-operation and Development Creditor Reporting System database. Where possible, we checked these amounts against the funding data available from government aid agencies. Estimates of multilateral ODA financing for HIV/AIDS were based on the country allocations announcement by the Global Fund to Fight AIDS, Tuberculosis and Malaria (the Global Fund) for the period 2014 to 2016.

**Results:**

Countries in the Asia-Pacific region receive the largest share of aid for HIV from the Global Fund. Bilateral funding for HIV in the region has been relatively stable over the last decade and is projected to remain below 10% of the worldwide response to the epidemic. Bilateral donors continue to prioritize ODA for HIV to other regions, particularly sub-Saharan Africa; Australia is an exception in prioritizing the Asia-Pacific region, but the United States is the bilateral donor providing the greatest amount of assistance in the region. Funding from the Global Fund has increased consistently since 2005, reaching a total of US$1.2 billion for the Asia-Pacific region from 2014 to 2016.

**Conclusions:**

Even with Global Fund allocations, countries in the Asia-Pacific region will not have enough resources to meet their epidemiological targets. Prevention funding is particularly vulnerable and requires greater domestic leadership and coordination. Bilateral donors are still crucially important in the response to HIV throughout the Asia-Pacific region.

## Introduction

**Figure 1:**
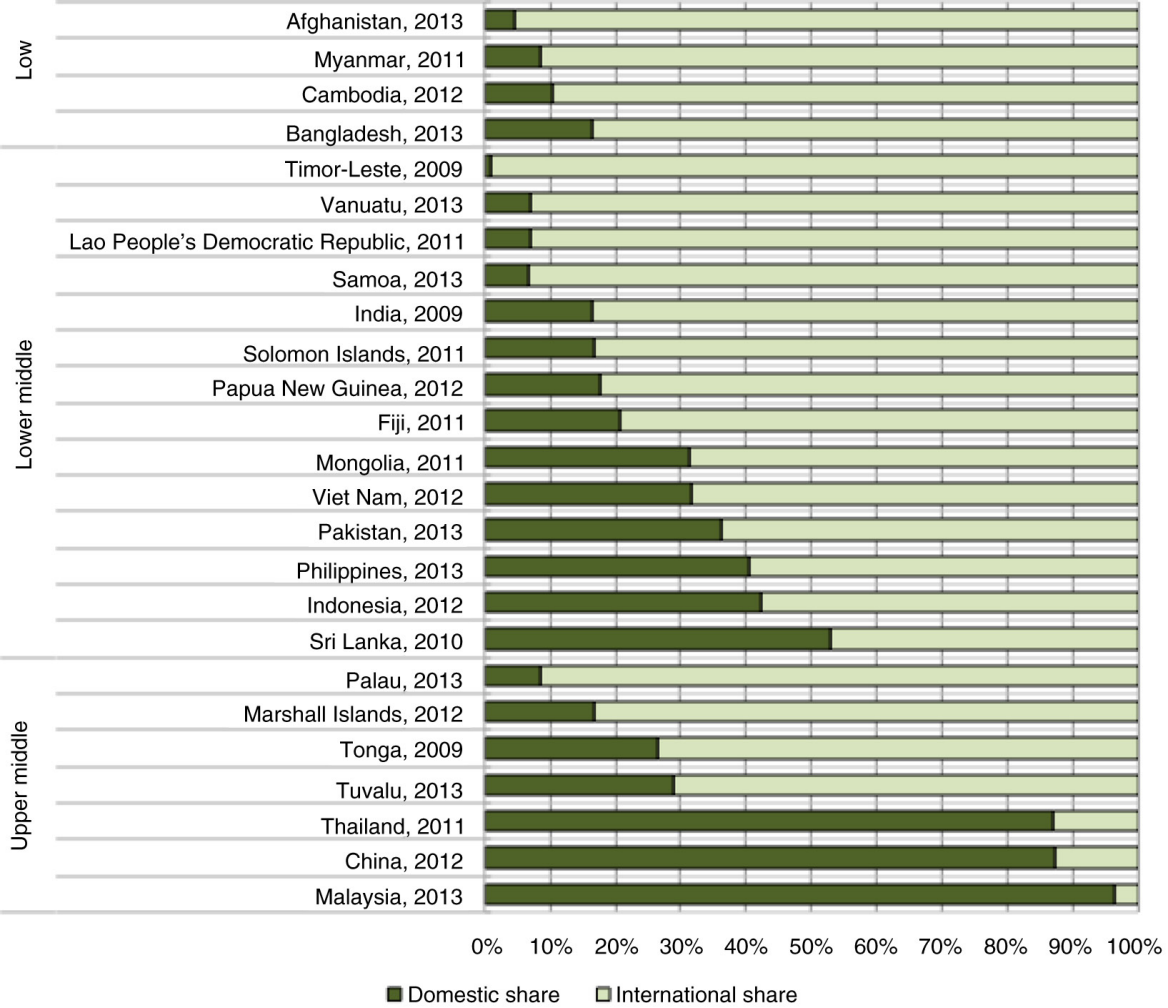
International versus domestic funding for HIV/AIDS programmes in Asia-Pacific, 2012. (Source: UNAIDS AIDS DataHub [www.aidsdatahub.org].)

All low-income and most lower-middle-income countries in the Asia-Pacific region are heavily reliant on international funding for HIV programmes (Figure 1). This dependence has persisted despite robust economic growth across Asia and the Pacific over the past decade. However, there are likely to be significant cuts to international funding for HIV programmes in the region following the adoption of a new funding model by the region's largest donor, the Global Fund to Fight AIDS, Tuberculosis and Malaria (the Global Fund). The new model uses epidemiological and economic criteria to determine funding eligibility. A number of countries in Asia and the Pacific are above or will soon rise above the Global Fund's threshold of $2000 gross national income per capita; they will therefore receive less funding from the Global Fund in the future. This new funding model reflects an expectation on governments in the Asia-Pacific region to be less reliant on external aid and invest more of their own resources in the health and wellbeing of their populations [1].

It is important to estimate not only how much official development assistance (ODA) for HIV will be available for countries in the Asia-Pacific region but also the likely sources of this funding. This study assesses the outlook for external assistance for HIV in the Asia-Pacific region.

## Methods

We sought to describe the availability of bilateral and multilateral (Global Fund) ODA for HIV programmes in the Asia-Pacific region. The recipient countries considered in this analysis are Afghanistan, Bangladesh, Bhutan, Cambodia, China, Fiji, India, Indonesia, Kiribati, Korea, Laos, Malaysia, Maldives, Marshall Islands, Micronesia, Mongolia, Myanmar, Nepal, Pakistan, Palau, Papua New Guinea, Philippines, Samoa, Sri Lanka, Solomon Islands, Thailand, Timor-Leste, Tonga, Tuvalu, Vanuatu and Vietnam. These 31 countries together make up the World Bank's definition of the low- and middle-income countries in the East Asia, Pacific and South Asia regions, which we refer to as the *Asia-Pacific region*.

To estimate multilateral and bilateral ODA financing for HIV between 2004 and 2013, we searched the Organisation for Economic Co-operation and Development (OECD) Creditor Reporting System (CRS) database for funding data in Subsectors 13040 (STD control including HIV/AIDS) and 16064 (Social mitigation of HIV/AIDS). Where possible, we checked these amounts against the funding data available from government aid agencies. To estimate multilateral ODA financing for HIV/AIDS for 2014 to 2016, we used the country allocations announced by the Global Fund for this period, with health-system-strengthening funding excluded. Given that the Global Fund has provided a significant share of multilateral HIV financing in the Asia-Pacific region, we take its funding allocations as a proxy for the total amount of multilateral funding that will be available. For comparative purposes, and to understand whether HIV still represents an ODA priority for donor countries, we also estimated country donor contributions to the Global Fund that are HIV-attributable. We obtained estimates of donor contributions between 2005 and 2013 and pledges for 2014 to 2016 from the Global Fund's website [2].

## Results

### International aid for HIV in the Asia-Pacific region, 2004 to 2013

#### Major sources of international aid for HIV

**Figure 2:**
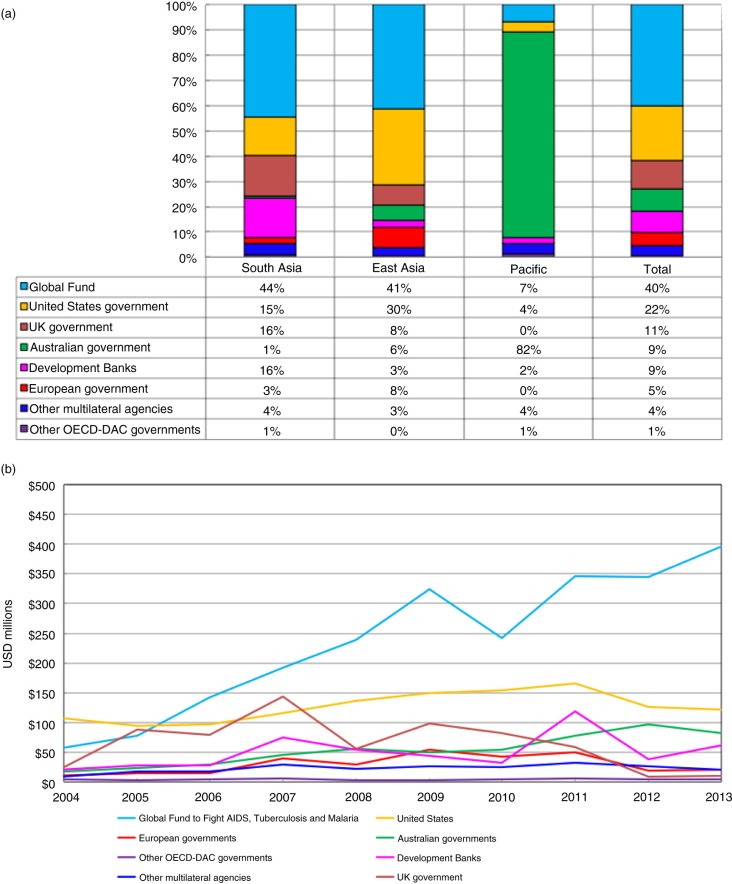
Trends and growth in international aid for HIV in the Asia-Pacific region. (a) The major sources of international aid for HIV in the Asia-Pacific region, 2004 to 2013. (Source: OECD-CRS database.) (b) Growth in international aid for HIV in the Asia-Pacific region, 2004 to 2013. (Source: OECD-CRS database.)

The largest international contributors to HIV programmes in the Asia-Pacific region between 2004 and 2013 were the Global Fund and the US government, which together provided over 60% of all international funding to the region (Figure 2a, final bar). The remaining ~40% of international aid for HIV in the region came from the UK government, the Australian government, development banks, European and other OECD Development Assistance Committee governments and other multilateral agencies. The primary sources of funding differ by subregion, with the Pacific receiving ~80% of its funding from the Australian government, while South and East Asia each receive ~80% from the Global Fund and the US and UK governments (Figure 2a, first three bars). Globally, the share of aid for HIV provided bilaterally is around three-quarters ($6.4 billion annually) [3]; between 2004 and 2013, the bilateral share of aid to South and East Asia was lower than the global average (36 and 53%, respectively), but much higher than the global average in the Pacific.

The total share of funding provided by the US government and the Global Fund remained relatively stable over the decade, but the share provided bilaterally by the US government decreased from 42% in 2004 to 17% in 2013, with this decline offset by increases in the Global Fund share (from 23 to 55%). The relative importance of the US government as a donor is reinforced by the fact that they were responsible for between 30 and 45% of the annual total pledges to the Global Fund between 2001 and 2016; adjusting for this, we estimate that around one-third of HIV funding to the region was contributed by the US government, either indirectly or directly.

#### Growth in international aid for HIV

Annual international funding for HIV in the Asia-Pacific region increased by an average of ~10% per annum between 2004 and 2013, with almost all of this annual average increase (nine percentage points) attributable to the growth in Global Fund allocations (Figure 2b). Allocations from other development banks and multilateral agencies remained essentially flat over this period. Increases in bilateral ODA from Australia contributed two percentage points to the total growth in international funding over the decade to 2013, while decreases from the UK government subtracted one percentage point.

#### Bilateral donors’ preferences and patterns

Between 2004 and 2013, ~99% of bilateral funds for HIV disbursed in the Asia-Pacific region and recorded in the CRS database originated from 10 donor countries: the United States, the United Kingdom, Australia, Germany, Sweden, Norway, Canada, the Netherlands, France and Japan. In 2013, these 10 countries allocated US$238 million to the Asia-Pacific region. In the same year, these 10 countries pledged US$3060 million to the Global Fund, of which an estimated 9%, or US$280 million, was allocated to HIV programmes in the Asia-Pacific region. All countries except Australia committed more to HIV programmes in the region via their Global Fund contributions than they did bilaterally.

**Figure 3:**
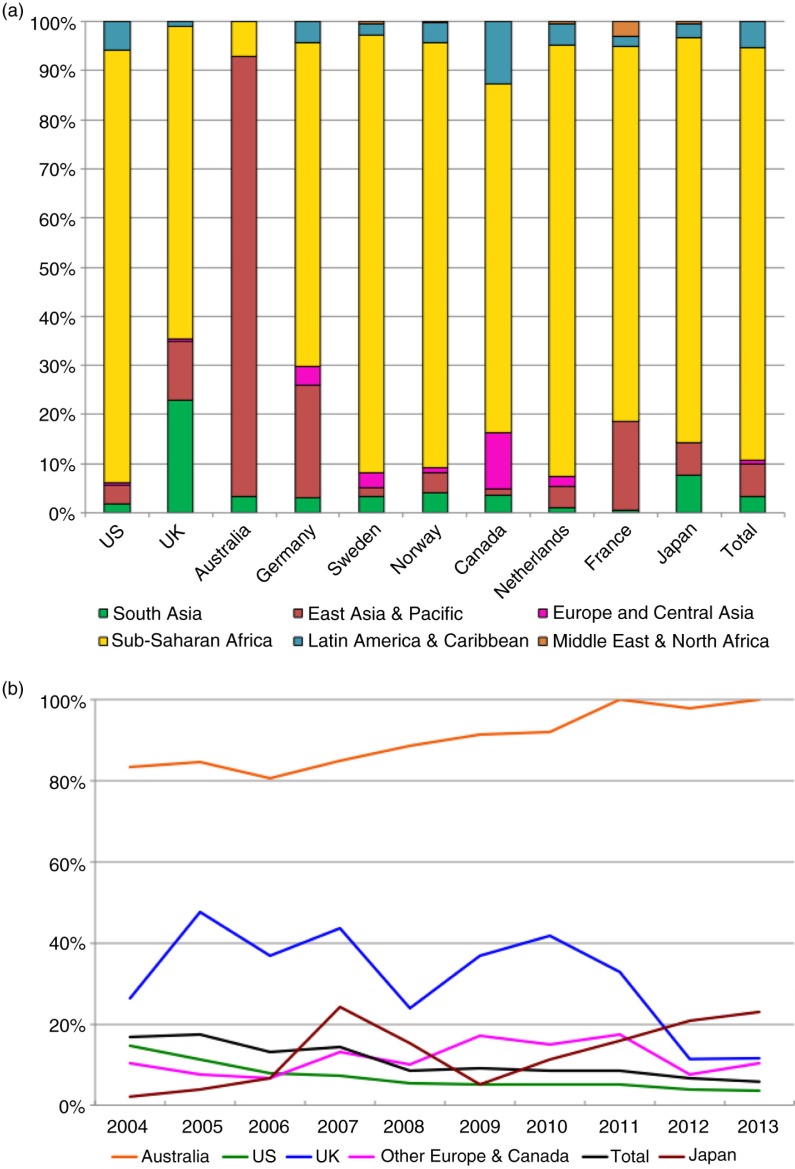
Bilateral donors’ preferences and patterns. (a) Bilateral funding for HIV by global region, 2004 to 2013. (Source: OECD-CRS database.) (b) The share of bilateral funding for HIV allocated to the Asia-Pacific region, 2004 to 2013. (Source: OECD-CRS database.)

Of the 10 most significant bilateral donors in the Asia-Pacific region, all except Australia contribute far more to countries in other regions than they do to countries in the Asia-Pacific. Just under 85% of all bilateral ODA for HIV from these 10 countries was allocated to sub-Saharan Africa, compared to ~10% allocated to the Asia-Pacific region (Figure 3a). The proportion of all bilateral funding allocated to the Asia-Pacific region has been steadily decreasing between 2004 and 2013, from 17% in 2004 to 6% in 2013, mostly driven by declines in the share allocated to the region by the regions’ two largest bilateral donors, the United States and the United Kingdom. However, several countries, including Australia, Germany, Japan and the Netherlands, increased the proportion of their total HIV aid budgets allocated to the region over this period (Figure 3b).

#### International aid for HIV per person infected

**Figure 4:**
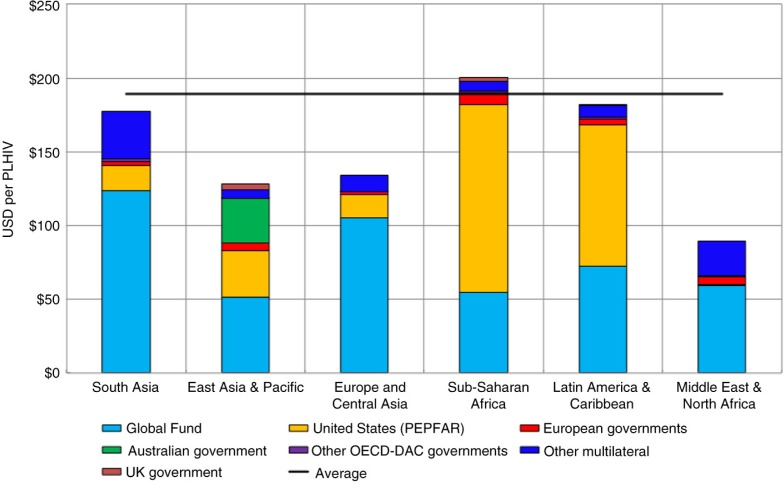
International aid for HIV per PLHIV, 2013. (Sources: OECD CRS database; UNAIDS.)

In order to estimate whether the distribution of ODA for HIV is roughly in line with the distribution of the epidemic, we calculate the amount of ODA for HIV per person infected for each region (Figure 4). While the number of PLHIV is only a very rough indicator of the epidemic burden, we can nevertheless observe that the distribution of total ODA is more even once the scale of the epidemic has been taken into account. The Asia-Pacific region received approximately US$150 per PLHIV in 2013, compared to a global average of US$190.

#### How international aid for HIV is spent

**Figure 5:**
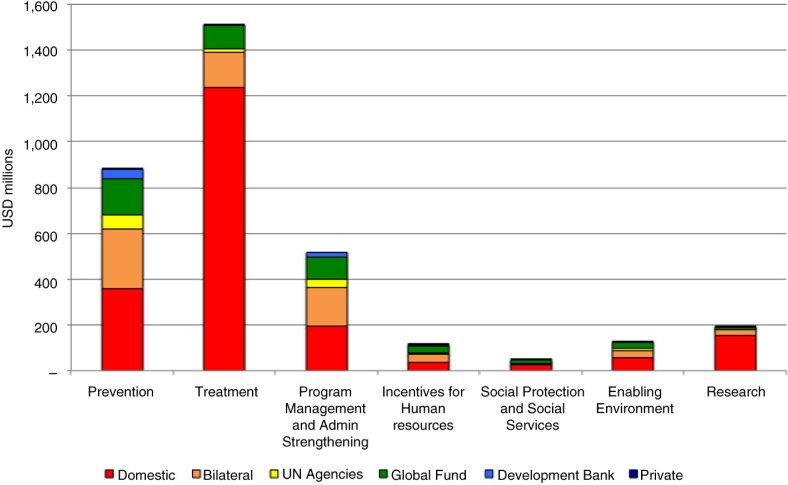
Allocation of funding among HIV programs in the Asia-Pacific region, 2005–2012. (Source: Global AIDS Response Progress Reporting database)

We used the UN's Global AIDS Response Progress Reporting database to investigate how funds allocated for HIV were spent over the period from 2005 to 2012 (Figure 5). International aid is particularly important for funding prevention programmes, with around 60% of prevention funding attributable to international donors. Other areas that are heavily dependent on international aid include programme management and administration strengthening (~60% international) and incentives for human resources (~65% international). The region as a whole is relatively self-sufficient when it comes to funding treatment programmes, with ~80% of treatment funded domestically.

#### Aid for HIV and total health aid

The results of a 2008 WHO investigation into the CRS database found that funding for HIV/AIDS accounted for almost one-third (32%) of total health ODA for the period 2002 to 2006. We found that this proportion has not decreased significantly since then, with funding for HIV/AIDS accounting for 30% of total health ODA between 2007 and 2013.

### Aid for HIV in the Asia-Pacific region in 2014 and beyond

#### Global Fund allocations for HIV

HIV-attributable donor pledges to the Global Fund reached US$12 billion in the fourth allocation (2014 to 2016) round, representing a 30% increase over the previous round. All donor countries have increased their contributions to the Global Fund since 2005, with most of the increases attributable to previous commitments and current pledges by the United States. Global Fund allocations for HIV have increased across all world regions, including a fourfold increase in funding from the Global Fund for HIV programmes in the Asia-Pacific region since 2005.

The Global Fund has currently allocated 16% of total funding excluding existing health-system-strengthening funds to the Asia-Pacific region. This represents a total of US$1.2 billion over the three-year allocation period, 2014 to 2016, with all low- and middle-income countries in Asia remaining on the Global Fund's eligibility list in the fourth replenishment round. Countries in sub-Saharan Africa receive the vast majority (69%) of Global Fund allocations.

#### Bilateral allocations for HIV

We previously identified 10 bilateral donors that contributed >99% of funds for HIV over the past decade. We examined the national budgets of nine of these 10 countries (budgets for Germany and Japan were not available at the time of data collection) and estimate that a total of US$204 million in ODA for HIV was budgeted the Asia-Pacific region in 2014, more than half of which is attributable to the United States. These nine governments pledged over six times this amount (US$1.3 billion) to the Global Fund.

Beyond 2014, some of the larger contributors of HIV funding to the Asia-Pacific region have already or are expected to decrease their ODA budgets. While Australia's assistance has increased in recent years, Australia's overall fiscal policy and strategic foreign policy direction mean that international assistance for HIV will likely stagnate or decrease over the next several years [4]. The United States and Netherlands are also expected to reduce their ODA budgets for HIV through to 2016.

## Discussion

Across the Asia-Pacific region, governments will require continued ODA for HIV to respond to their national HIV epidemics and achieve epidemiological targets [5,6]. There is already a large resource gap between current HIV funding and the level required in order to bring about targeted reductions in HIV-related incidence and mortality in Asia [6,7]. Trends in ODA for HIV suggest that this gap is likely to widen.

While bilateral funding for HIV at a global level has increased in previous years, donors continue to concentrate ODA for HIV to countries in sub-Saharan Africa. Pledges to the Global Fund and continued bilateral ODA for HIV in sub-Saharan Africa suggest that HIV still represents a funding priority for donors. However, bilateral ODA for HIV in the Asia-Pacific region has remained close to constant over the decade to 2013 and is a relatively low priority among most bilateral donors. Bilateral donors are already decreasing funding for HIV programmes in middle-income countries across the Asia-Pacific region for future years due to governments’ intentions to refocus HIV funding and fiscal austerity measures following the global financial crisis [5]. While most donors have displayed a continued preference for providing HIV assistance bilaterally, most countries in the Asia-Pacific region can continue to expect to receive the majority of external assistance for HIV multilaterally. The 2014 OECD Development Assistance Committee survey on donors’ forward-spending plans indicated that overall (HIV- and non-HIV-related) programmed aid to low-income countries in Africa and elsewhere will decline. The survey also suggests a continued donor focus in the medium term on middle-income countries, including some projected increases in programmable aid for middle-income countries across the Asia-Pacific region. However, much of these increases are likely to take the form of soft loans and are unlikely to be HIV-specific [8].

The Global Fund's model of allocating funding to HIV according to epidemiological and economic criteria means that all low- and middle-income countries in the region currently remain eligible for HIV funding. A number of countries in the Asia-Pacific region – including the three largest, China, Pakistan and Indonesia – are presently among the Fund's designated “highest impact” subset, for which Global Fund investments are believed to have the highest impact on outcomes [9]. Yet there is considerable uncertainty about whether countries will remain eligible for Global Fund resources past 2016 as their economic growth continues.

While there is currently no consensus on what constitutes a reasonable domestic contribution to HIV responses [10,11], governments of recipient countries are now expected by the international community to transition towards self-sufficiency in financing their HIV programmes to align with economic growth across the region [5]. There have already been significant steps in this direction within the Asia-Pacific region: between 2004 and 2013, the number of people in the region accessing antiretroviral therapy increased from 70,000 to 1,250,000, and an estimated 80% of these treatment costs were funded from domestic sources. However, despite high-level political commitment to funding HIV responses [5], most national governments across the Asia-Pacific region have expressed uncertainty on the question of where to find additional funding for HIV within their domestic budgets. HIV prevention programmes for key affected populations, which up until now have been primarily funded by donors, are particularly at risk [12].

An important concern for providers of international aid is the extent to which international aid to governmental sectors might crowd out the government and private sectors in some countries as the government reallocates their funds to other priority areas. This concept of aid displacement (or fungibility) implies that donor funds intended for health are effectively used to fund other things. The average fungibility of aid for HIV has not been well established, with various publications reporting conflicting messages [13,14]. Competing domestic budgetary priorities across the health sector and political agendas mean that domestic resources will not automatically replace declining ODA [6,15]. This issue is an open question into which further research would be very welcome.

Although national governments should continue to make progress towards achieving domestic ownership of their HIV responses [16], it is clear that the transition to domestic funding will require a phased approach [5], including provisions that respond to countries’ existing politics and policies that affect governments’ abilities to bridge financial gaps. Consideration of the policy environment in recipient countries has not tended to be greatly influential in donor aid allocation decisions [17–19]. However, policies and laws against the often marginalized members of key affected populations at higher risk of HIV infection must be addressed to facilitate effective prevention programmes [20]. This action is especially important because these critical programmes are the most vulnerable to being dismantled as they are currently mainly funded from international sources. Although it does not explicitly consider the political environment of recipient countries, the Global Fund's use of qualitative criteria to adjust aid allocation decisions within its new funding model, including past programme performance, risk and absorptive capacity [21], are positive steps in this direction. However, the Global Fund's emphasis on funding to achieve the greatest programme impact [22] means that there is still likely room for improvement in balancing considerations of efficiency and equity [23].

The experience of low national HIV prevalence and recent economic growth among countries in the Asia-Pacific region does not preclude their continued need for donor assistance, at least in the short term. Until the principle of health for all [24] can be fully realized on the political and budgetary agendas of governments across the Asia-Pacific region, countries will need donors’ continued fiscal support for HIV. In addition to the total amount of aid available for HIV, the mode of aid disbursement has important implications for aid effectiveness [25,26]. The increased use of soft loans in the future may mean middle-income Asian and Pacific countries may expect more ODA funding in general in the future [8]. However, the impact of this on HIV financing is uncertain. Despite some weaknesses [27,28], the shift towards multilateral disbursement of ODA for HIV may help to overcome some of the challenges in achieving aid harmonization [29,30] that can result from having multiple sources of bilateral aid [31]. This has become a pertinent issue in Asia as the number of bilateral donors has proliferated over the last decade [32]. Potential advantages of multilateral disbursement of ODA include increased harmonization of aid flows and enhanced collective action for HIV in the region [33,34].

The current study reviewed trends in bilateral and multilateral aid for HIV in the Asia-Pacific region. However, it is important to note that this analysis did not include all relevant multilateral and bilateral donors in the Asia-Pacific region. In particular, data for China were not available at the time of data collection. The statistics on international HIV-specific assistance reported by the OECD CRS do not include all forms of international assistance. In addition, the CRS data may not include certain funding streams provided by donors, such as HIV components of mixed grants to non-governmental organizations.

## Conclusions

Countries in Asia-Pacific are increasingly taking leadership for the care and treatment of their populations of people living with HIV, as evidenced by increased coverage of antiretroviral therapy and increased proportional funding contributions from domestic sources. However, most countries in the region are heavily reliant on international sources, particularly the Global Fund and PEPFAR (complemented by bilateral sources), for their HIV prevention programmes. Specifically, prevention programmes for people most at risk of acquiring HIV are almost entirely funded from international sources. Domestically funded prevention programmes are all too often non-targeted or targeted at general lower risk populations [15]. With all of the accumulated knowledge and evidence, and especially considering the future financing landscape, domestically funded HIV programmes need to deliver effective prevention strategies that focus on key populations and are designed to respond to local conditions and epidemiology. Developing and implementing these prevention programmes demands strong domestic leadership and may ultimately lead to better coordination of the HIV response in the longer term. However, in the medium term, the Global Fund and bilateral donors will remain crucially important to the HIV response in the Asia-Pacific region.
